# Bio-Based and Nanostructured Polymers for Sustainable Protection of Cultural Heritage and Medicinal Crops: Convergence of Heritage Science, Circular Bioeconomy, and Environmental Protection

**DOI:** 10.3390/polym17192582

**Published:** 2025-09-24

**Authors:** Irina Fierascu, Anda Maria Baroi, Roxana Ioana Matei, Toma Fistos, Irina Elena Chican, Cristina Emanuela Enascuta, Sorin Marius Avramescu, Radu Claudiu Fierascu

**Affiliations:** 1 National Institute for Research & Development in Chemistry and Petrochemistry–ICECHIM Bucharest, 202 Spl. Independentei, 060021 Bucharest, Romania; irina.fierascu@icechim.ro (I.F.); anda.baroi@icechim.ro (A.M.B.); roxana.brazdis@icechim.ro (R.I.M.); toma.fistos@icechim.ro (T.F.); irina-elena.chican@icechim.ro (I.E.C.); cristina.enascuta@icechim.ro (C.E.E.); 2 Faculty of Horticulture, University of Agronomic Science and Veterinary Medicine, 59 Marasti Blvd., 011464 Bucharest, Romania; 3 Faculty of Animal Productions Engineering and Management, University of Agronomic Sciences and Veterinary Medicine of Bucharest, 59 Marasti Blvd., 011464 Bucharest, Romania; sorin.avramescu@usamv.ro; 4 Faculty of Chemical Engineering and Biotechnology, National University of Science and Technology Politehnica Bucharest, 1-7 Gheorghe Polizu St., 011061 Bucharest, Romania; 5 Academy of Romanian Scientists, 3 Ilfov, 050044 Bucharest, Romania

**Keywords:** bio-based polymers, nanostructured polymers, cultural heritage conservation, medicinal and aromatic plants, sustainable agriculture, environmental protection, circular economy, smart polymers, chitosan, nanocellulose

## Abstract

Polymers have long been central to modern materials science, but their durability has also made them major contributors to environmental pollution. A new generation of bio-based and nanostructured polymers is now reshaping this field, offering materials that are functional, reversible, and sustainable. This review examines their role across three interconnected domains: cultural heritage conservation, the protection of medicinal and aromatic plants (MAPs), and environmental sustainability. In heritage science, polymers are moving away from synthetic resins toward renewable systems such as chitosan, nanocellulose, and PLA, which provide stability while remaining reversible and compatible with delicate substrates. In agriculture, biodegradable coatings, controlled-release carriers, and edible films are improving MAP protection, extending shelf life, and reducing reliance on synthetic pesticides. In environmental applications, polymers are being reinvented as solutions rather than problems—through degradable mulches, functional hydrogels, and nanocomposites that clean soils and waters within a circular economy framework. Looking across these domains reveals strong synergies. The same principles—biodegradability, multifunctionality, and responsiveness—apply in each context, turning polymers from passive barriers into intelligent, adaptive systems. Their future success will depend not only on chemistry but also on life-cycle design, policy alignment, and public trust, making polymers key enablers of sustainability.

## 1. Introduction

Polymers have become indispensable to modern materials science, shaping progress in fields as varied as packaging, medicine, structural engineering, and electronics. Their strength lies in their vast variety, as well as their extraordinary versatility: by adjusting chemical composition, molecular architecture, processing conditions, or physical properties, scientists can create materials that meet almost any functional need [1]. In recent decades, this versatility has extended far beyond industrial applications. Polymers now play an important role in areas that touch directly on society’s most pressing concerns, such as the preservation of cultural heritage, the sustainable cultivation of agricultural resources, and the protection of natural ecosystems [2].

In these contexts, polymers are not simply used as passive supports or structural materials. They are engineered to be active, responsive, and multifunctional, able to adapt to complex environments and solve specific problems. This ability to combine functionality with adaptability makes them particularly relevant in an era when science and technology are expected to deliver solutions that are not only effective but also safe for the environment and aligned with social responsibility [3].

In the field of cultural heritage conservation, polymers have long been valued as consolidants, adhesives, protective coatings, and cleaning agents [4]. From the mid-twentieth century onwards, synthetic polymers such as poly(vinyl acetate), acrylic resins, and epoxies became widely adopted thanks to their ease of application and apparent stability [5]. Over time, however, these materials revealed their limitations: many underwent yellowing or embrittlement, or proved incompatible with delicate historical substrates [6]. Even more problematic was the issue of irreversibility—once applied, these polymers were difficult, if not impossible, to remove without risking damage to the original material [7].

Today, the conservation community is rethinking its reliance on traditional synthetic polymers. There is a growing demand for materials that are not only functional and durable but also reversible, minimally invasive, and environmentally responsible [8]. This has opened new opportunities for bio-based and nanostructured polymers, which can be tailored to provide precise functionalities while respecting the ethical and sustainability principles that guide modern conservation practices.

A similar story is unfolding in agriculture, where polymers are increasingly recognized as valuable tools for improving efficiency and sustainability [9]. Conventional agrochemicals often suffer from poor stability, rapid degradation, and unintended environmental consequences [10]. By contrast, polymers can be designed as protective coatings, controlled-release matrices, or encapsulation systems that deliver active ingredients more effectively and with less environmental burden [11].

This is particularly important for medicinal and aromatic plants (MAPs)—crops with high economic and pharmacological value but notable sensitivity to pests, pathogens, and post-harvest deterioration. Polymers can reduce the frequency of pesticide applications by releasing bioactive agents gradually and precisely where needed, thereby lowering environmental contamination [12]. When these polymers are derived from renewable resources such as chitosan, cellulose, starch, or polylactic acid, their use directly supports the transition toward a circular bioeconomy in which agricultural residues and biomass are revalorized as feedstocks for new functional materials [13].

Environmental protection represents another domain where polymers are proving indispensable [14]. The success of synthetic polymers has created one of today’s most visible ecological crises: the persistence of plastics in soils, waterways, and even living organisms. Microplastics, in particular, have become emblematic of the long-term consequences of designing materials without considering their end-of-life [15].

However, polymers are also at the heart of potential solutions. Functional polymeric adsorbents can capture heavy metals or organic pollutants from contaminated water [16]. Membranes based on advanced polymer structures can purify industrial effluents [17]. Biodegradable films and coatings can replace single-use plastics, reducing waste accumulation [18]. The real frontier now lies in designing polymers that are both highly effective in their protective functions and intrinsically degradable or recyclable, thereby closing the material loop and significantly lowering their environmental footprint.

What is striking is how much these three domains—cultural heritage, agriculture, and environmental protection—have in common when viewed through the lens of polymer science. Each faces the challenge of moving away from traditional synthetic polymers toward bio-based and nanostructured alternatives that combine high performance with sustainability. Each requires materials that are multifunctional, compatible with sensitive biological or cultural substrates, and resilient under environmental stress. Together, they illustrate the growing role of polymers as a cross-cutting technology that bridges sectors and fosters interdisciplinary innovation.

This shift toward sustainable polymer systems is strongly reinforced by global policy frameworks [19]. The European Green Deal calls for climate neutrality by 2050, with emphasis on resource efficiency, pollution reduction, and circular economy strategies [20,21]. The United Nations Sustainable Development Goals (SDGs) link responsible consumption and production (SDG 12), climate action (SDG 13), protection of ecosystems and biodiversity (SDG 15), and the safeguarding of cultural heritage as part of resilient, sustainable communities (SDG 11) [22,23].

In this global context, polymers play a paradoxical role. On the one hand, synthetic polymers—especially those derived from fossil resources—are a major contributor to environmental degradation. On the other, well-designed bio-based and biodegradable polymers can become essential tools for achieving sustainability goals, helping societies to preserve heritage, protect crops, and restore natural ecosystems.

The circular bioeconomy provides a particularly powerful framework for rethinking polymer development. Unlike the linear model of “take–make–dispose” that has dominated materials production for decades, the bioeconomy promotes renewable resources, efficient use of biomass, and reintegration of materials into biological or technical cycles at end-of-life [24]. Bio-based polymers such as polylactic acid, polyhydroxyalkanoates, lignin derivatives, and polysaccharides are prime examples. Produced from agricultural residues or microbial fermentation, they not only reduce dependence on fossil carbon but also create direct synergies with the agricultural sector. When nanostructured—through techniques such as electrospinning, nanoprecipitation, or self-assembly—these polymers can achieve properties that exceed those of conventional materials, such as high surface area, tunable porosity, and stimulus-responsive behavior.

For cultural heritage, nanostructured bio-based polymers can penetrate more deeply into porous substrates, remain optically transparent, and release protective agents in a controlled way [25]. For medicinal and aromatic plants, they can encapsulate volatile compounds, stabilize antioxidants, and deliver natural pesticides in a slow and sustained manner, maximizing efficacy while minimizing residues [3]. For environmental protection, nanostructured polymers can act as highly efficient adsorbents, catalysts for pollutant degradation, or self-degrading protective films [14,26,27]. In all cases, sustainability is not an afterthought, but a core principle of design.

This trend is also driven by societal expectations. Communities expect cultural heritage to be preserved in ways that respect ecological responsibility. Consumers of MAP-derived products demand clean, residue-free cultivation. Citizens around the world increasingly reject materials that leave behind a legacy of waste and pollution. The move toward sustainable, bio-based, and nanostructured polymers is therefore not only a scientific and technical necessity, but also a response to public demand and ethical responsibility.

In this critical review, it is examined the way how bio-based and nanostructured polymers can act as a unifying platform across cultural heritage conservation, medicinal and aromatic plant protection, and environmental sustainability. Will be discussed the role of polymers in cultural heritage, from early synthetic consolidants to emerging green alternatives, their applications in MAP cultivation and preservation, highlighting controlled release, encapsulation, and post-harvest strategies developed within a circular bioeconomy framework, and, finally, will be presented the environmental dimension: both the challenges posed by persistent plastics and the solutions offered by bio-based and degradable polymers in remediation and pollution control.

Throughout, it will be emphasized the synergies that emerge when these fields are considered together. Conservation science’s focus on reversibility and substrate compatibility can inform agricultural polymer design. Advances in controlled release for MAPs can inspire novel approaches to long-term protection of artworks. Environmental research on polymer degradation and fate provides a shared knowledge base for predicting material performance in both agricultural and heritage contexts.

Ultimately, the aim of the present work is not only to review current progress but also to identify future directions for research at the intersection of polymer chemistry, nanotechnology, conservation, agro-ecology, and environmental engineering. The development of bio-based, nanostructured polymers can be considered more than a technical endeavor: it is a cultural and ecological necessity, reflecting the new expectations placed on materials science in the twenty-first century.

## 2. Polymers at the Crossroads of Heritage, Agriculture, and Environment

Polymers have accompanied human societies throughout the last two centuries, reshaping technologies and material practices in ways that were unimaginable before their discovery. Their entrance into fields such as cultural heritage conservation and agriculture was greeted with enthusiasm: they seemed to offer durable, versatile, and affordable solutions where natural materials—like gums, glues, or waxes—had long proven fragile or inconsistent [6]. For a time, polymers lived up to this promise. They allowed conservators to strengthen ancient objects and farmers to protect crops with modern efficiency. Yet the enthusiasm was tempered over time, as the drawbacks of synthetic polymers became more visible. Poor reversibility in conservation, ecological persistence, and their contribution to microplastic pollution revealed the hidden costs of a materials strategy that prioritized short-term function over long-term sustainability.

The present era marks a turning point. The rise in bio-based polymers, coupled with powerful nanostructuring techniques, reflects not just a technological advance but a philosophical shift in material design. It is a move toward performance balanced with ecological responsibility and societal values.

### 2.1. A Historical Perspective: Synthetic Polymers in Heritage Conservation and Agriculture

The mid-twentieth century witnessed the rapid adoption of synthetic polymers such as poly(vinyl acetate) (PVAc), acrylic resins, epoxies, and polyurethanes. Each family of materials carried technical advantages that made them attractive to very different but equally demanding domains: cultural heritage conservation and agriculture.

In cultural heritage, polymers were embraced as modern, scientific alternatives to traditional materials. Conservators, who for centuries had relied on animal glues, natural gums, or waxes, saw in synthetic polymers an opportunity to achieve greater stability and reliability. Acrylic resins, for example, became popular consolidants for porous stones and wall paintings: they could penetrate deeply into substrates, solidify into transparent films, and seemed chemically stable [28,29]. Paraloid™ B-72 (Rohm and Haas, for example), presented in the 1950s, became, quickly became a reference consolidant for stone, paintings, and ceramics [30]. Its optical transparency and solubility in mild solvents contributed to its widespread use, although long-term studies later revealed yellowing and embrittlement. Epoxy resins, with their exceptional adhesive strength, allowed broken ceramics, glass, or stone to be rejoined with unprecedented durability [31,32]. Resins such as Araldite^®^ AY103/HY956 were extensively used in the 1960s–80s for rejoining ceramics, glass, and stone fragments [33]. Their exceptional bond strength, however, made them practically irreversible. PVAc emulsions, such as Vinavil^®^ 59 or Mowilith^®^ DMC2, already widely used in the paper and textile industries, were repurposed as adhesives in conservation [34,35], taking advantage of their initial flexibility and low cost, while polyurethanes appeared in foams and protective coatings [36].

In agriculture, polymers found roles that paralleled those in heritage but with a different emphasis. PVAc and acrylic copolymers were used to coat seeds and fertilizers, improving adhesion, stability, and controlled release [37]. Epoxy-based formulations were applied as protective coatings for agricultural machinery and packaging [38]. Polyurethanes, particularly Desmophen^®^/Desmodur^®^ foams, were employed to protect wood, agricultural equipment, and in mulching applications [39]. During the 1960s and 1970s, with the intensification of the Green Revolution, synthetic polymers were increasingly integrated into the formulation of pesticides, herbicides, and fertilizers, acting as carriers, adjuvants, or slow-release coatings [40,41,42].

**Figure 1:**
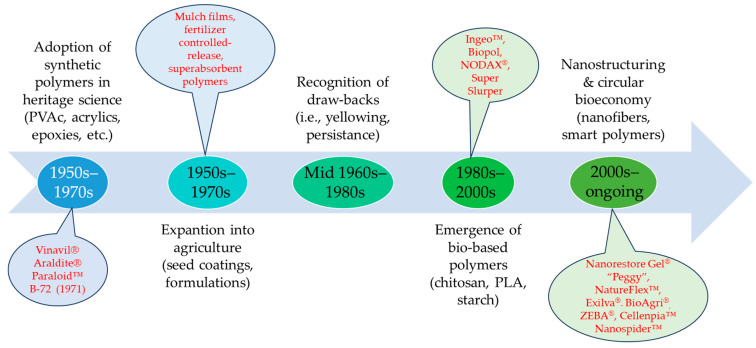
Timeline of polymer use in heritage and agriculture.

These examples show how synthetic polymers became not only research curiosities but standardized commercial products across heritage and agriculture within two decades. Their adoption was based on perceived inertness and durability, but this very durability later created compatibility and environmental issues. The adoption of these polymers was driven by a belief in their chemical inertness and durability. In conservation, “inert” meant non-reactive with valuable substrates, while in agriculture it implied resistance to weathering. For decades these assumptions held, and polymers were celebrated as modern materials that could extend the life of cultural artifacts, enhance agricultural yields, and deliver reliable performance (Figure 1).

### 2.2. The Challenges of Traditional Polymeric Materials

As synthetic polymers became ubiquitous, their limitations began to surface. These challenges fall broadly into three categories: lack of reversibility and compatibility, environmental persistence, and pollution, including microplastics.

#### 2.2.1. Lack of Reversibility and Incompatibility in Heritage Conservation

Reversibility is a cornerstone of conservation ethics: any intervention should be removable without damaging the original artifact [43]. Synthetic polymers often fell short of this principle. Epoxy resins, once cured, proved almost impossible to remove, frequently leaving behind residues that darkened or stained substrates [44,45]. Acrylic consolidants, initially clear, sometimes yellowed or embrittled with age, causing irreversible visual and mechanical alterations [46]. Even PVAc emulsions, which were thought to be stable, often cross-linked over time, losing solubility and making removal difficult [34]. For example, the effect of different adhesives (belonging to the main classes commonly used in ceramic conservation: epoxies, acrylics and cellulose nitrate formulations) on architectural ceramic tiles (*azulejos*) was evaluated by Musacchi et al. [47]. Their performance was investigated both in terms of mechanical behavior and in response to artificial and natural aging, and the results highlight clear contrasts between the categories. The three epoxies—Hxtal NYL-1 (HXTAL Adhesive, Coconut Grove, Australia), Fynebond (Fyne Conservation Services, Argyll, Scotland) and Araldite 2020 (Huntsman, TX, USA)—exhibited consistently high stiffness and strength. Dynamic mechanical analysis showed glass transition temperatures ranging roughly between 44 and 55 °C in the as-cured state, shifting upward to around 60–70 °C after climatic aging, a sign of further crosslinking. Flexural testing confirmed that these adhesives bonded the tiles so strongly that the ceramic itself fractured before the joint did, demonstrating that their cohesive and adhesive strength was greater than the flexural strength of the ceramic substrate. Although this outcome might appear positive from a purely mechanical perspective, it presents a serious drawback in conservation practice, since it means that under stress the historic ceramic will fail before the join, producing new fractures. Epoxies also showed a tendency to yellow after aging and to undergo chemical alterations; moreover, their high viscosity and short working time make them less user-friendly during application. The acrylic resins produced more moderate results. Paraloid B72 (Rohm and Haas, Philadelphia, PA, USA) emerged as the best-balanced option among them. With a glass transition temperature close to 40 °C, it remained sufficiently flexible, and in flexural tests it produced mixed adhesive–ceramic failures that suggest a bond compatible with the mechanical characteristics of the substrate. Importantly, B72 maintained or even improved its strength after both artificial and natural aging, and it showed low yellowing. Its main weaknesses are linked to its solvent-rich nature: because it is used as a solution with roughly 60% solvent, it undergoes shrinkage as the solvent evaporates and may develop bubbles in the joint; it can also soften under elevated temperature, around 55 °C, which was evident in chamber tests. By contrast, Paraloid B44 (Rohm and Haas, Philadelphia, PA, USA), with a lower glass transition near 30 °C, gave weaker joins, predominantly adhesive failures, and its performance declined after aging. In addition, its relatively low viscosity at the concentrations tested led to excessive absorption into the porous ceramic, complicating bonding. Paraloid B67 (Rohm and Haas, Philadelphia, PA, USA) behaved even more poorly: it was brittle, produced very weak bonds that frequently failed adhesively, and after aging most samples disintegrated before mechanical testing could even be performed. The cellulose nitrate adhesives—represented by UHU Hart (UHU^®^ GmbH & Co., Baden, Germany), HMG Cellulose Nitrate (HMG Paints Ltd., Manchester, UK), and Archaocoll 2000 (Kremer Pigmente Gmbh & Co., Aichstetten, Germany)—performed worst overall. They initially provided medium strength in the case of UHU Hart, comparable to B72, with mixed adhesive–ceramic failures. However, all the cellulose nitrate formulations were already somewhat yellow in their as-prepared state and underwent marked yellowing and chemical alteration after aging. Mechanical degradation was severe: some aged samples were so fragile that they could not be subjected to dynamic mechanical analysis. Flexural strengths decreased substantially after aging, with most failures occurring adhesively. Among them, Archaocoll 2000 and HMG CN showed the lowest stability, while even UHU Hart, the relatively stronger member of the group, lost performance with time. Taken together, the study reveals that epoxies, despite their excellent strength, are unsuitable for historical ceramics because their mechanical superiority translates into a high risk of creating new breaks, and because they suffer from yellowing and limited reversibility. Cellulose nitrates show poor stability, marked yellowing and chemical alteration, and cannot be considered reliable over the long term. Only Paraloid B72 provided a satisfactory compromise, with good compatibility, relative stability and manageable application properties, although even it has drawbacks linked to solvent evaporation and softening under heat. These findings reinforce the principle that in conservation of ceramics, adhesives should not simply maximize bond strength but rather provide a balance between adhesion, stability and compatibility with the fragile, historic substrate [47]. Karayannidou et al. [33], presented a kinetic and material-science investigation into the epoxy–amine systems most frequently adopted by conservators for joining glass and ceramic artifacts. The authors focused on two commercial systems that had become standards in conservation practice: Araldite 2020, a two-component bisphenol-A-based epoxy cured with a cycloaliphatic polyamine hardener, and the AY103/HY956 system, another widely used epoxy–amine formulation. The study applied Fourier-transform infrared spectroscopy (FT-IR) to monitor the curing reactions of these adhesives in real time, thereby characterizing their cure kinetics under different conditions. Both systems showed the typical behavior of amine-cured epoxies, where the reaction proceeds rapidly at first and then slows down as the mobility of the reactive groups decreases with network formation. The researchers observed that Araldite 2020 in particular achieved a high degree of conversion, indicating that under proper mixing and curing conditions it forms a dense crosslinked network. This dense structure explains its very high mechanical strength and durability, properties that make it appealing for ceramic and glass restoration because it can hold joins securely even under load. However, the same property also poses the main drawback: the adhesive bond is often mechanically stronger than the ceramic itself, so under stress the artifact is more likely to fracture elsewhere rather than at the join, creating new and irreparable damage. The AY103/HY956 system also produced a rigid, crosslinked network, though its cure kinetics were slightly different, with a somewhat slower overall conversion compared to Araldite 2020. Both adhesives were transparent in their freshly cured state, a major advantage for esthetic reasons when restoring translucent or glazed ceramics. Nevertheless, the authors noted that aging—particularly thermal and photochemical exposure—can induce yellowing and some chemical changes in these systems, as had already been reported by other conservation studies. Another limitation is their irreversibility: once cured, these adhesives are extremely difficult to remove without damaging the underlying ceramic. In addition, their relatively high viscosity compared to solvent-based acrylics can make them challenging to apply in fine or porous joins, particularly when delicate alignment of fragments is required [33].

Regarding the application of PVAc adhesives, Novak et al. [35] performed a comprehensive review of PVAc in heritage paints., noting that hydrolysis of PVAc produces PVOH and acetic and that photodegradation induces cross-linking and gel formation leading to partial insolubility (classic observation: PVAc becoming “partially insoluble in benzene” after UV, attributed to cross-linking). Chelazzi et al. [48] also presented a study on PVAc adhesives used in conservation, reporting spectroscopic changes (incl. FTIR) consistent with deacetylation (ester hydrolysis) and network changes upon aging; the authors also observed and discusses increased gel/insoluble fractions associated with cross-linking—directly supporting the insolubility aspect after prolonged aging.

Beyond reversibility, mechanical incompatibility created further problems. The stiffness, elasticity, or thermal expansion of synthetic polymers often differed significantly from the materials they were meant to protect—be it stone, wood, or plaster. These mismatches could generate stresses that led to cracking, delamination, or accelerated deterioration [49]. What seemed at first to be inert, protective solutions sometimes created new risks for the very artifacts they were meant to preserve.

#### 2.2.2. Environmental Persistence and Waste Accumulation

In agriculture, similar issues emerged but with environmental rather than ethical implications. Polymer coatings and carriers often remained in the soil long after their useful life [50]. Plastic mulching films, irrigation pipes, and protective coverings fragmented into ever smaller pieces rather than degrading, adding to environmental pollution. Even in conservation, the disposal of polymer-based materials from restoration treatments and laboratory waste streams contributed to plastic accumulation [51].

Durability, once celebrated, became a double-edged sword. Resistance to microbial attack meant that many synthetic polymers persisted for decades or centuries, accumulating in soils, waterways, and marine systems [52,53].

#### 2.2.3. Microplastics and Toxicological Concerns

The fragmentation of persistent polymers has given rise to the now-familiar crisis of microplastics, defined as particles smaller than 5 mm [54]. Their sources are diverse: weathered agricultural films, degraded seed coatings, worn packaging materials, and deteriorated conservation coatings. Even though the conservation field contributes relatively small volumes, the global impact is magnified by the vast use of polymers in agriculture and other industries.

Microplastics not only persist physically but also interact chemically with their environment. They can carry toxic additives, plasticizers, or adsorbed pollutants, acting as vectors for environmental contamination. Their ingestion by soil organisms, aquatic life, and potentially humans raise serious health concerns [55]. Tumwesigye et al. [56], in example, discusses how microplastics persist physically in environments and interact chemically by adsorbing pollutants, toxins and carrying chemical additives. They examine evidence that microplastics can accumulate heavy metals, persistent organic pollutants (POPs), pharmaceuticals, and other harmful chemicals, and that microplastics can act as vectors, transferring these contaminants to soil, aquatic organisms, and potentially up the food chain. The growing awareness that polymers designed for stability could become long-term environmental hazards has fueled the search for alternatives that marry performance with ecological responsibility.

### 2.3. The Rise in Bio-Based Polymers as Sustainable Alternatives

In response to these challenges, attention has turned to bio-based polymers—materials derived from renewable biological resources such as plants, animals, and microorganisms. Unlike synthetic polymers derived from fossil carbon, bio-based polymers are integrated into natural cycles and often biodegradable, making them attractive as sustainable alternatives [57].

#### 2.3.1. Chitosan

Chitosan, derived from the deacetylation of chitin (abundant in crustacean shells or produced by fungi), is a versatile, cationic polysaccharide with film-forming, biocompatible, and antimicrobial properties [58]. In agriculture, it is used to coat seeds, fruits, and leaves, reducing fungal infections and extending post-harvest shelf life [59]. In cultural heritage, chitosan-based coatings have shown promise in protecting stone and wood against microbial colonization [60,61]. Its biodegradability into non-toxic sugars makes it a particularly attractive alternative to persistent synthetics.

#### 2.3.2. Cellulose Derivatives

Cellulose—the most abundant polymer on Earth—has a long history in conservation, especially in paper and textile restoration [62]. Modern derivatives such as carboxymethyl cellulose (CMC), hydroxypropyl cellulose (HPC), and nanocellulose expand its applications. These materials are soluble, film-forming, and transparent, making them suitable as consolidants and coatings. Nanocellulose, in particular, offers high mechanical strength, tunable rheology, and unique optical properties [63]. In agriculture, cellulose-based hydrogels improve soil water retention [64], while in environmental protection they act as biodegradable adsorbents for pollutants [65].

#### 2.3.3. Starch

Starch, derived from crops like corn, potatoes, or rice, is inexpensive and renewable. It can be processed into thermoplastic starch or chemically modified for improved stability. In agriculture, starch-based materials serve as carriers for fertilizers and pesticides, allowing controlled release [66]. In conservation, starch pastes remain widely used for paper restoration [67], and modified starches are being tested for broader consolidant and coating functions [68]. Its ready biodegradability is a key advantage, though sensitivity to humidity can be a limitation.

#### 2.3.4. Polylactic Acid (PLA)

PLA is a biopolyester synthesized by fermenting sugars into lactic acid followed by polymerization. It is one of the flagship bio-based polymers, widely studied as a replacement for fossil-derived plastics. PLA offers good mechanical properties, processability, and compatibility with nanostructuring techniques. In agriculture, PLA nanoparticles can encapsulate pesticides or fertilizers for controlled release [69]. In conservation, PLA-based nanocomposites have been investigated as protective coatings for stone and metal [70]. While PLA biodegrades most effectively under industrial composting conditions, its renewable origin and adaptability make it a valuable sustainable option.

#### 2.3.5. Lignin

Lignin, a major by-product of the paper and biofuel industries, is often burned as low-value fuel but has significant potential as a polymer feedstock. Its complex aromatic structure gives it natural UV-absorbing and antioxidant properties. In agriculture, lignin-based carriers can encapsulate fertilizers or pesticides, protecting active ingredients from photodegradation [71]. In conservation, lignin-containing blends are investigated as coatings that can mitigate UV damage to sensitive artifacts [72]. The valorization of lignin within a circular bioeconomy also adds economic and environmental value.

#### 2.3.6. Proteins

Proteins such as gelatin, casein, and soy protein have been used for centuries in adhesives, paints, and coatings. Today, they are being re-examined as sustainable polymeric materials. Their ability to form transparent films and to interact chemically with diverse substrates makes them attractive for conservation adhesives or coatings. In agriculture, protein-based coatings can be applied to seeds and fruits as biodegradable protective layers [73]. Their sensitivity to moisture and microbial attack can be a drawback in some cases, but in contexts where biodegradability is desired, these same properties are advantageous [74].

Together, these bio-based polymers provide a diverse toolkit that can replace or complement synthetic materials, offering biodegradability, renewability, and functionality consistent with the principles of the circular bioeconomy. A synthetic comparison between synthetic and bio-based polymers across domains is presented in Table 1.

### 2.4. Nanostructuring: Enhancing Performance Through Design

Although bio-based polymers are inherently sustainable, they sometimes fall short of synthetic counterparts in mechanical strength, stability, or controlled release capabilities. Nanostructuring techniques offer a solution by enhancing their performance and enabling new functionalities [79].

#### 2.4.1. Nanofibers

Electrospinning and related methods can produce polymer nanofibers with diameters from tens to hundreds of nanometers. These fibers exhibit high surface area-to-volume ratios, tunable porosity, and the ability to incorporate active agents [80,81]. In conservation, electrospun nanofibers have been developed as breathable, transparent coatings or carriers for antimicrobial agents [82]. In agriculture, nanofiber mats can be loaded with pesticides or fertilizers, ensuring gradual release and reduced leaching [83]. When produced from biodegradable polymers such as PLA or chitosan, nanofibers combine functionality with environmental responsibility [84].

#### 2.4.2. Nanocapsules and Nanoparticles

Encapsulation at the nanoscale offers a way to protect sensitive bioactives—such as essential oils, phenolic compounds, or natural pesticides—from volatilization, oxidation, or photodegradation. Nanocapsules made from starch, PLA, or chitosan allow slow and targeted release. In agriculture, this enables pest control with minimal environmental residues [85,86,87]. In conservation, nanoparticles of consolidants can penetrate deep into porous materials, strengthening them without altering their visual appearance [88,89].

#### 2.4.3. Nanocomposites

By combining bio-based polymers with inorganic nanoparticles (such as silica, titanium dioxide, or clays), researchers create nanocomposites with superior properties. In conservation, nanocomposites provide both mechanical reinforcement and optical transparency, ideal for fragile stone or plaster [25]. In agriculture, they regulate the release of fertilizers and offer UV shielding [9]. In environmental protection, nanocomposites are engineered as adsorbents or photocatalysts capable of removing or degrading pollutants [16,90].

#### 2.4.4. Smart and Responsive Polymers

Perhaps the most exciting frontier is the development of stimuli-responsive polymers, capable of changing their properties in response to environmental triggers such as pH, humidity, temperature, or light. For example, protective coatings could “breathe” in response to humidity, opening pores to release antimicrobials only when moisture fosters microbial growth [4]. In agriculture, delivery systems could release bioactives only under specific soil pH or after rainfall, improving efficiency and reducing waste [91]. This responsiveness mirrors the complexity of real-world environments, making smart polymers an area of growing interest.

Table 2 presents a short overview of the applications of emerging nanostructured polymer systems (nanofibers, nanocapsules, nanocomposites, smart polymers).

### 2.5. Polymeric Materials at the Crossroads

The historical use of synthetic polymers demonstrated just how profoundly polymer science could transform domains as diverse as cultural heritage conservation, agriculture, and environmental protection. For conservators, they provided tools to consolidate fragile substrates and to bond fractured artifacts in ways that had not been possible before [104]. For agronomists, they offered a means of enhancing crop resilience, controlling the delivery of agrochemicals, and increasing yields in the context of an expanding global population [105]. For engineers and environmental scientists, they seemed to embody modernity itself: lightweight, strong, and versatile [106].

Yet this very success also revealed the hidden risks of a design philosophy centered on durability without degradability, on strength without reversibility, and on performance without ecological foresight. In heritage contexts, polymers that were once hailed as inert often proved to be incompatible or irreversible, effectively “locking in” past interventions and creating new conservation dilemmas. In agriculture, the persistence of plastic mulches, coatings, and carriers resulted in the accumulation of residues in soils, contributing to the rise in microplastic pollution. In environmental contexts, the narrative of polymers as problem-solvers became overshadowed by the recognition that they were also a primary source of long-lasting pollutants.

Today, a new era is emerging, defined by bio-based and nanostructured polymers that combine renewable origins with enhanced functionalities [75,107,108]. This shift is not merely a matter of substituting one material with another; it signals a broader transformation in the values that underpin material design. Modern polymer science increasingly embraces life-cycle thinking, in which the origin, performance, and end-of-life of a material are all considered part of a coherent sustainability framework. This is evident in the development of biodegradable consolidants for heritage, controlled-release agricultural coatings derived from renewable feedstocks, and smart, self-degrading polymers for environmental protection.

At this crossroads, polymers are viewed through a dual lens: as a major environmental challenge when designed without consideration of their fate, and as one of the most promising enablers of sustainability when designed with ecological foresight. The paradox of polymers, once a source of frustration, can now be reframed as an opportunity. By confronting the legacy of persistence and incompatibility, researchers are reimagining polymers not as inert “fixes” but as dynamic systems that respond to environmental stimuli, degrade when their function is complete, and integrate harmoniously into natural or circular cycles.

This transition is also reinforced by broader societal and political frameworks. Policies such as the European Green Deal and the United Nations Sustainable Development Goals have catalyzed research and industrial investment in bio-based materials [109,110]. At the same time, public awareness of microplastics and plastic pollution has created social pressure for change. As a result, the materials community faces not only scientific challenges but also an unprecedented alignment of incentives that favor sustainable innovation.

The crossroads metaphor is therefore apt: polymer science stands between the legacy of the twentieth century and the aspirations of the twenty-first. On one path lies the continued reliance on fossil-derived, persistent plastics with mounting ecological costs; on the other lies the opportunity to craft a new generation of polymers that are renewable, biodegradable, multifunctional, and adaptable. The outcome will not depend on chemistry alone, but on the willingness of scientists, conservators, agronomists, policymakers, and industry to adopt a systems perspective—seeing polymers not in isolation, but as integral parts of cultural, ecological, and economic cycles.

In conclusion, the trajectory of polymers from synthetic dominance to bio-based innovation illustrates more than a technological evolution: it reveals a fundamental reorientation of material science toward responsibility and resilience. The next decades will determine whether polymers can transform from symbols of environmental crisis into cornerstones of a sustainable future. Their success in this role will depend on interdisciplinary collaboration, continued nanotechnological refinement, and the embedding of ethical and ecological principles at the very heart of polymer design.

## 3. Polymeric Systems for Cultural Heritage Protection

Cultural heritage represents one of the most delicate and demanding domains for polymer science. Here, the stakes extend well beyond material performance: every intervention affects artifacts that are unique, irreplaceable, and often centuries old. This means that the materials chosen to stabilize, consolidate, or protect them must satisfy requirements more rigorous and specific than those applied in industry. At the same time, conservation is governed by ethical principles of reversibility, minimal intervention, and sustainability, which place additional constraints on the use of modern materials [111].

Against these restraints, polymers have played a dual role. They have been indispensable tools for protecting cultural assets, but in some cases have also introduced new long-term problems. Synthetic resins once celebrated for their strength and transparency later proved incompatible, irreversible, or environmentally persistent. Today, a new generation of bio-based and nanostructured polymers is offering the possibility of treatments that are at once technically effective, reversible, and environmentally responsible.

### 3.1. Requirements of Conservation Polymers

The requirements for polymers in conservation arise not only from technical needs but also from ethical considerations. A consolidant or adhesive used in industry may be judged sufficient if it is strong and durable. In cultural heritage, by contrast, performance must be balanced with sensitivity to historical materials, esthetic integrity, and future reversibility.

Stability and durability. Conservation polymers must withstand the stresses of real environments: fluctuating temperature, humidity, exposure to light, and atmospheric pollutants. Acrylic resins, for instance, once thought to be stable, were later observed to yellow and embrittle [5], undermining the very works they were intended to protect. Importantly, stability must be proven not only under laboratory conditions but also in complex heritage environments such as underground fresco chambers, where humidity accelerates polymer aging.

Reversibility. A defining principle of modern conservation is that interventions should be undoable, leaving open the possibility of future re-treatment. This principle is underscored by the negative legacy of early epoxy repairs on ceramics—irreversible bonds that trapped fragile fragments in ways that modern conservators can no longer correct [47]. By contrast, bio-based polymers like chitosan can be dissolved or enzymatically broken down [2], offering genuine pathways to reversibility.

Optical transparency. Many heritage materials—paintings, manuscripts, textiles, stone carvings—derive much of their value from appearance. A polymer consolidant for paper must strengthen fibers without producing gloss or clouding. A protective coating for stone must prevent water infiltration without altering surface color. Here, nanocellulose has shown promise: applied to fragile archival paper, it can improve tear resistance while preserving flexibility and transparency [62].

Compatibility. Polymers must be mechanically and chemically compatible with the substrate. Mismatches can generate stresses that lead to cracking or delamination, as seen when PVAc was applied to wooden panels whose thermal expansion differed from the adhesive [112]. Cellulose derivatives, by contrast, harmonize naturally with paper and textile fibers [113], reflecting chemical similarity.

Non-toxicity and sustainability. Safety is critical not only for conservators but also for museum staff, visitors, and the cultural object itself. In addition, the conservation community is now embracing green conservation materials—renewable, biodegradable, and produced with minimal environmental impact. Chitosan, starch, and PLA exemplify this shift, showing how polymers can combine technical performance with sustainability. For example, chitosan has been used successfully to inhibit salt crystallization damage to limestone while remaining compatible with the stone’s properties [114] or has been applied for microbial protection of painting surfaces in cultural heritage objects [60]. Meanwhile, PLA-starch laminates have shown good stability and compostability in environmental exposure, demonstrating that combining PLA with starch can maintain technical performance over time for different applications [115].

In sum, the requirements for conservation polymers extend well beyond functionality. They encompass a complex matrix of technical reliability, esthetic neutrality, reversibility, and ecological responsibility, reflecting the unique challenges of safeguarding cultural heritage.

### 3.2. Bio-Based Polymers in Conservation

The limitations of traditional synthetic resins have driven interest in bio-based polymers, derived from renewable sources such as plants, animals, or microorganisms. These materials are biodegradable, often more compatible with natural substrates, and sometimes provide intrinsic protective functions.

Among bio-based polymers, chitosan has emerged as a leading candidate because of its antimicrobial activity, which arises from its positively charged molecular structure that disrupts microbial cell membranes [116]. This makes it effective against fungi and bacteria that colonize stone, wood, and mural surfaces. For example, chitosan, used at 1% concentration, was proven an effective solution for painting providing protection against mold fungi, without any significant effect on the spectral and surface properties of sturgeon glue [117]. In archeological wood, chitosan solutions penetrated into cell walls, improving cohesion and resistance to biodeterioration [118]. Unlike many synthetic biocides that leave insoluble residues, chitosan can be redissolved in mild acidic solutions, enhancing reversibility.

Cellulose, already the main constituent of paper and textiles, has long been central to conservation. The development of nanocellulose—whether as nanofibrils (CNF) or nanocrystals (CNC)—has opened new applications. Nanocellulose suspensions have been applied to archival paper, restoring mechanical strength while maintaining transparency and flexibility [119]. In stone conservation, bacterial nanocellulose hydrogels have been used as green cleaning agent for cleaning of copper stains from marble [120]. Such versatility illustrates how bio-based polymers can serve both as consolidants and as innovative cleaning systems.

Proteins (such as gelatin) were historically used as adhesives, but fell out of favor due to susceptibility to microbes and moisture. Today, nanotechnology is reviving them. Gelatin nanoparticles, synthesized using a two-step desolvation method, where proposed for consolidation of gelatin-based photograph emulsions [121]. Recent studies have explored silk fibroin as a consolidation agent for aged silk artifacts, exploiting its ability to form films with tunable internal structures. By adjusting the concentration of fibroin dispersions, the crystallinity of the resulting films can be modulated, which directly affects the mechanical behavior of treated fibers. Concentrated dispersions, richer in crystalline domains, tended to reduce fiber elongation, whereas lower concentrations, with greater amorphous character, improved flexibility and mechanical performance. The most effective treatments were obtained with dilute dispersions, which restored tensile strength close to that of pristine silk while enhancing elongation [122].

Starch remains a mainstay in paper conservation, often used to attach Japanese repair tissues. Modified starches with enhanced water resistance are now tested for broader applications, such as strengthening textiles or even serving as carriers for protective additives [123,124]. A recent proteomics-based study has clarified the adhesive properties of starch- versus flour-based glues in conservation. Wheat starch paste, long considered a cornerstone of book and paper restoration, owes its functionality to amylose and amylopectin, which form a flexible hydrogen-bonded network with porous materials. Unlike flour pastes, which are rich in gluten proteins and thus prone to microbial degradation, starch pastes exhibit chemical simplicity and stability, producing durable and reversible bonds that do not cross-link over time. Importantly, proteomic analysis revealed that starch pastes contain significantly fewer protein components than flour-based ones, which not only explains their greater stability but also makes them less likely to interfere with future analytical investigations. These findings confirm why starch paste continues to be the adhesive of choice for hinging, mending, leafcasting, and other key conservation practices, despite its somewhat lower adhesive strength compared with flour glue [125]. PLA (polylactic acid), more commonly associated with packaging, is now being adapted into conservation coatings. In a comprehensive study on stone protection, bio-nanocomposite coatings based on poly-L-lactide (PLA) reinforced with montmorillonite (MMT) nanoclays were applied to Marmara marble. These coatings significantly improved surface hydrophobicity, with contact angles reaching over 108° at 5 wt% clay loading, while reducing both capillary water absorption and water vapor permeability. Importantly, they provided strong protection against SO_2_-induced sulfation, reducing gypsum crust formation by up to five times compared with untreated marble. At optimal concentrations (2–5 wt%), the coatings maintained the marble’s color and appearance, fulfilling a central conservation requirement. However, excessive filler content (7 wt%) led to poor dispersion, slight color alteration, and diminished performance. The biodegradable nature of PLA further suggests reversibility, positioning PLA/MMT nanocomposites as promising candidates for sustainable and effective marble conservation [70]. The use of PLA in this context exemplifies how industrial bio-based materials can be re-engineered to serve conservation needs.

Together, these examples demonstrate that bio-based polymers are not merely “green replacements” for synthetics. They represent a new philosophy of conservation materials—one in which renewable origin, biodegradability, and substrate compatibility are integral design criteria, rather than afterthoughts. Table 3 provides a comparative overview of their main applications, using representative examples, advantages, and challenges, offering a concise reference for their role in meeting the demanding requirements of heritage preservation.

### 3.3. Nanostructured Polymeric Systems in Heritage Science

Nanostructuring allows conservation polymers to achieve a new level of performance, enabling them to penetrate deeper, act more selectively, and respond to environmental triggers.

At the nanoscale, encapsulation improves delivery and control. An example of bio-based nanostructured coatings recently developed for fresco conservation is represented by system that uses a chitosan hydrogel containing in situ synthesized silver nanoparticles, coupled with azelaic and lactic acids in a zero-waste one-pot synthesis. The formulation achieves brushable rheology via a glycerol-ethanol solvent mixture. In tests against established commercial products (Paraloid B72, Proconsol^®^), the hybrid coating demonstrated high chemical stability, minimal changes in color or gloss, maintained surface morphology, hydrophobic behavior, and controlled vapor permeability—highlighting its potential as a sustainable, effective, and esthetically compatible coating for outdoor wall paintings [125]. A particularly innovative area of research has focused on chitosan nanoparticles as carriers for natural biocides, overcoming the volatility and short-lived efficacy of essential oils. In one recent study, thymol—a component of thyme essential oil with known antifungal and antibacterial properties—was successfully encapsulated into chitosan nanoparticles using an emulsification–ionic gelation process [126]. The resulting nanocarriers, with spherical morphologies around 80–100 nm, exhibited enhanced thermal stability, controlled release, and improved antimicrobial activity compared with free thymol. Laboratory tests showed that thymol-loaded nanoparticles inhibited Aspergillus niger growth at lower concentrations than either free thymol or blank chitosan particles, while release kinetics confirmed a sustained diffusion of thymol for up to 30 days in aqueous conditions. The system was validated in an on-site application at the Feilaifeng limestone heritage site (China), where areas treated with thymol-loaded nanoparticles displayed a 74% reduction in microbial ATP levels after three months, compared with only 58% for free thymol and negligible effects for blank chitosan [126]. These results demonstrate that nanostructured chitosan carriers can transform short-lived natural antimicrobials into long-lasting, eco-friendly protective treatments, reducing the frequency of interventions while maintaining compatibility with sensitive stone surfaces.

Electrospinning produces nanofiber mats that are highly porous, breathable, and optically transparent [127]. In a notable example of sustainable nanostructured protection, Camargos et al. [128] formulated coatings combining cellulose nanofibrils (CNFs), cellulose nanocrystals (CNCs), and lignin nanoparticles (LNPs). These nanocomposites offered robust protection against moist-heat aging and UV exposure, thanks to LNP-derived antioxidant properties, while preserving surface morphology and maintaining vapor permeability. Coatings remained optically transparent, and when topped with carnauba-wax nanoparticles, achieved water contact angles up to 120°. Crucially, the system was fully reversible and composed entirely of biodegradable, renewable components—demonstrating a genuinely green solution for treating cellulose-based heritage substrates like paper, textiles, and wood [128]. Another example of nanostructured polymer systems for conservation is provided by the development of SiO_2_–fluorinated PLA bionanocomposites designed as reversible and highly hydrophobic coatings for building stones [129]. By combining a fluorinated polylactide copolymer with silica nanoparticles, researchers created a nanocomposite coating capable of producing water contact angles of ~140°, significantly enhancing hydrophobicity through the synergistic effect of low surface energy (from fluorine groups) and increased surface roughness (from dispersed silica particles). When applied to marble, the coatings effectively reduced capillary water uptake by up to 98% while maintaining water vapor permeability within acceptable limits for conservation (less than 50% reduction compared to untreated stone). Importantly, chromatic alterations remained below perceptible thresholds (ΔE* < 3), preserving the esthetic integrity of the substrate. The treatment also proved reversible, as coatings could be removed by simple solvent application (chloroform), a crucial requirement in heritage conservation [129]. These results demonstrate how PLA-based nanocomposites can be engineered into multifunctional protective systems that are not only biodegradable and renewable but also effective and reversible in practical applications to heritage stone. By combining permeability, transparency, and functional loading, electrospun fibers embody the principle of multifunctional, minimally invasive protection.

Perhaps the most exciting frontier lies in stimuli-responsive polymers, capable of adapting their behavior to environmental conditions. Recent research has highlighted hydrogels as one of the most promising classes of stimuli-responsive polymers for cultural heritage protection. Their high water content and flexible polymer networks allow them to be engineered to respond dynamically to environmental triggers such as humidity, pH, or temperature. In conservation, this responsiveness translates into systems that can release antifungal agents only under favorable conditions for microbial growth, or swell in damp environments to provide local humidity control. Some experimental hydrogel formulations have also been designed to be self-dissolving, ensuring complete reversibility once their protective role is fulfilled. Beyond microbial control, these systems can provide gentle structural stabilization and surface protection, all while leaving minimal residues on the artifact. Such adaptive hydrogels exemplify the direction of next-generation conservation materials: minimally invasive, reversible, and capable of acting “on demand” rather than continuously [130]. These innovations point toward a future where conservation materials act not as static layers but as dynamic partners, adapting to the needs of fragile artifacts over time.

### 3.4. Discussion and Perspectives

The integration of bio-based and nanostructured polymers into cultural heritage conservation reflects more than a shift in technical practice; it marks a philosophical transformation in how materials are conceived, tested, and applied. Where once the emphasis lay on achieving durability and apparent inertness, the new paradigm insists on aligning materials with ethical principles, ecological sustainability, and long-term adaptability.

The examples provided above show that the new generation of polymers is capable of meeting functional demands while addressing long-standing concerns about reversibility and compatibility. Yet they also expose the tensions inherent in conservation practice. For example, a polymer that biodegrades too quickly may not provide adequate long-term protection, while one that is too durable risks repeating the mistakes of past synthetics [131]. This tension points to one of the central challenges of the coming decades: how to balance biodegradability with functional longevity in ways that respect both the artifact and its environment.

Another issue is reversibility in practice versus principle. While many bio-based polymers are technically reversible, the act of removing them from fragile substrates is not always straightforward [132]. Testing reversibility in controlled conditions does not always capture the complexity of aged, contaminated, or mixed-material heritage objects. Developing standardized protocols for reversibility testing, alongside international databases of material performance, could provide the evidence base needed to guide decision-making across museums, archives, and conservation laboratories.

Beyond technical questions, the conversation increasingly extends to ethics and public expectations. Cultural institutions are under growing pressure to demonstrate not only that they preserve heritage, but that they do so in ways consistent with environmental responsibility. The emergence of the “green museum” movement illustrates this trend: institutions are rethinking energy use, exhibition design, and materials in storage and conservation. Polymers, as visible and symbolic materials of modernity, are part of this story. Choosing bio-based and adaptive systems is therefore not only a scientific decision but a statement about how society values heritage in relation to sustainability.

The policy context further amplifies these pressures. The European Green Deal, the UN Sustainable Development Goals, and national circular bioeconomy strategies provide both incentives and funding frameworks for research into bio-based materials. Conservation, which was once viewed as a relatively insulated domain, is increasingly embedded in these wider agendas. This alignment presents opportunities for heritage science to attract support and visibility, but also responsibilities to meet the expectations of transparency, accountability, and cross-sector impact.

Looking ahead, several promising avenues can be envisioned:

Biomimetic design. Inspiration from natural protective systems—such as plant cuticles, insect wings, or nacre—could lead to polymers that combine hydrophobicity, UV protection, and self-healing in ways that traditional resins cannot. Biomimetic approaches could bridge the gap between durability and biodegradability [133].

Digital and AI-assisted conservation science. Advances in computational modeling and machine learning could help predict the aging of polymers under complex environmental conditions. Virtual “stress tests” could accelerate the selection of candidate materials and reduce reliance on decades-long empirical studies. AI might also be used to optimize formulations, balancing mechanical, optical, and degradability parameters in ways too complex for traditional trial-and-error approaches.

Citizen science and public engagement. Since many heritage sites are exposed to outdoor environments, local communities could be involved in monitoring the performance of conservation materials (e.g., observing microbial regrowth on treated stone or discoloration of coatings). This would democratize conservation science and provide large-scale, real-world datasets.

Circularity in conservation practice. The idea of a circular bioeconomy has not yet been fully applied to conservation. Imagine polymers derived from agricultural waste streams being used to preserve heritage artifacts, closing a symbolic and material loop between cultural memory and natural resources. At the end of their useful life, these polymers could be designed to degrade safely into non-toxic products that feed back into natural cycles.

These directions illustrate that polymers in conservation are no longer just “products” to be applied, but part of a systems approach that connects cultural values, ecological responsibility, and technological innovation. The shift from static, irreversible synthetics to dynamic, bio-based, and adaptive systems is not simply an improvement in materials; it is a redefinition of conservation itself, where material science becomes inseparable from sustainability and societal expectations.

In this light, polymers are more than tools for preserving the past [134]. They are mediators between heritage and the future, embodying the possibility of safeguarding cultural memory while also modeling the ecological responsibility demanded by the twenty-first century.

## 4. Polymeric Systems for Sustainable Protection of Medicinal and Aromatic Plants (MAPs)

Medicinal and aromatic plants (MAPs) are unlike staple cereals or industrial crops in both purpose and value. They are cultivated not for calories or bulk biomass, but for their secondary metabolites—essential oils, alkaloids, terpenes, flavonoids, and phenolic compounds—that have long underpinned the pharmaceutical, cosmetic, nutraceutical, and food industries. The economic and cultural weight of these crops is disproportionate to their cultivation area [135]. A few hectares of lavender, thyme, mint, rosemary, or chamomile may generate far more value than a much larger field of wheat, precisely because the market prizes purity and bioactivity over yield.

Yet this high value is also a source of vulnerability. Because MAPs depend on delicate and often volatile molecules, they are particularly susceptible to pests, pathogens, climate stress, and degradation during storage and transport. Even minor compositional changes in essential oils can influence not only market price but also therapeutic efficacy. For growers and processors, this means that preservation of quality is as critical as maximizing yield.

Here, polymeric systems emerge as powerful allies. Biodegradable coatings, encapsulation matrices, and nanostructured carriers can stabilize fragile natural products, deliver protection against biotic and abiotic stressors, and extend the usable life of harvested material. Importantly, when derived from renewable resources, these systems also reflect the broader imperatives of the circular bioeconomy and the European Green Deal, making them environmentally responsible as well as technically effective.

In this section, will be examined the main challenges of MAP cultivation and post-harvest handling, the roles that polymers already play, and the more advanced nanostructured solutions that are beginning to reshape this field.

### 4.1. Challenges in MAP Cultivation

#### 4.1.1. Pest and Pathogen Threats

MAPs are prone to attack by a broad range of fungi, bacteria, and insects. Fungal pathogens such as *Botrytis cinerea*, *Fusarium* spp., and *Aspergillus* spp. thrive under humid conditions, often devastating yields [136]. Insect pests—aphids, whiteflies, thrips, and leaf miners—cause both direct tissue damage and indirect quality losses by transmitting viruses or inducing stress that alters metabolite production.

The use of conventional pesticides in MAP cultivation is especially problematic. Residues do not simply represent an environmental risk; they can directly contaminate the target product—the essential oil or extract—undermining purity and consumer trust. For high-end applications such as aromatherapy, perfumery, or phytopharmaceuticals, even trace residues may be unacceptable. Growing consumer demand for clean-label, residue-free production adds further pressure to reduce synthetic chemical use. For MAPs, the challenge is thus sharper than in many other crops: disease and pest management must be achieved without compromising the very compounds that give these plants their value.

#### 4.1.2. Climate Stress and Post-Harvest Degradation

MAPs are often grown in regions most exposed to climate variability—Mediterranean, semi-arid, or mountain environments. Drought, heat waves, irregular rainfall, and shifting seasonality all affect growth and, critically, the biosynthetic pathways of secondary metabolites [137]. Plants may produce less oil, or oils of altered composition, reducing both quantity and quality.

After harvest, these challenges continue. Essential oils are volatile and readily lost through evaporation; they are also prone to oxidation, isomerization, or microbial degradation [138]. Phenolic compounds, although less volatile, are light- and oxygen-sensitive, often degrading during storage or transport [139]. Losses in post-harvest quality can be substantial, and because these crops are sold for their bioactivity, quality loss translates directly into economic loss.

#### 4.1.3. High Value of Essential Oils and Phenolic Compounds

Because MAPs are valued for specific compounds rather than bulk yield, they require precision protection. A lavender oil that falls outside accepted linalool–linalyl acetate ratios may be downgraded [140], and a thyme oil with degraded thymol content may lose therapeutic efficacy [141]. The precision required to stabilize and preserve these compounds explains why polymeric systems—capable of controlling microenvironments, slowing release, and shielding against degradation—are so promising in this sector.

### 4.2. Polymers in Crop Protection

One of the most effective approaches is encapsulation of volatile oils or plant-derived biopesticides in biodegradable polymer carriers. By entrapping molecules like thymol, carvacrol, eugenol, or citral within chitosan, PLA, or alginate matrices, researchers have achieved stability against evaporation and light and created controlled-release formulations that deliver consistent antimicrobial or insecticidal activity [142]. A compelling example of polymer-enabled crop protection involves thymol-loaded chitosan nanoparticles (TCNPs), which have been shown to eliminate the bacterial pathogen *Xanthomonas campestris* within 24 h at 500 µg/mL. Mode-of-action studies illustrate that TCNPs disrupt bacterial cell membranes, induce membrane depolarization, and trigger reactive oxygen species production leading to lipid peroxidation. Complementary FTIR and metabolomic analyses confirm substantial damage to bacterial lipids, proteins, carbohydrates, and nucleic acids. This multi-pronged attack underlines TCNPs as a green, targeted, and highly effective polymer-based system for protecting medicinal and aromatic plants against bacterial threats [143]. Similarly, carvacrol-loaded chitosan nanoparticles, synthesized via ionic gelation with an optimal 1:1.5 (*w*/*w*) chitosan/carvacrol ratio. Spectroscopic and microscopic characterization confirmed effective encapsulation and desirable nanoparticle morphology. Bioassays showed potent activity against *Candida* spp. in planktonic cultures, with *C. tropicalis* and *C. krusei* proving highly susceptible, though antibiofilm activity varied by strain. This work underscores how chitosan-based nanoformulations can potentiate natural monoterpenes—creating targeted, biodegradable, and efficacious antifungal systems for crop protection and storage [144].

Encapsulation also reduces phytotoxicity. Many essential oils are effective antimicrobials but can damage plant tissues at high concentrations. Encapsulated forms provide a gentler, more sustained release that protects MAPs without harming them [145].

Conventional sprays are notoriously inefficient: rain washes them away, sunlight degrades them, and volatilization reduces their persistence. Polymers offer controlled-release solutions. Starch-based matrices, for instance, have been tested as carriers for insect repellents, releasing them slowly into the crop environment [41]. For growers, this approach reduces both the number of applications and overall chemical inputs, aligning economic efficiency with sustainability.

For fresh MAPs such as mint, basil, or parsley, and for dried materials such as chamomile flowers or lavender buds, post-harvest coatings are increasingly important. Thin edible films of chitosan, alginate, or starch can suppress microbial growth [146], slow respiration, and reduce oxidation. An illustrative example comes from coriander, where chitosan nanoparticle (CSNP) edible coatings were shown to extend shelf life by more than twofold compared to untreated leaves. Beyond simply delaying wilting, CSNPs preserved key quality markers—including chlorophyll, carotenoids, proteins, and essential oils—while reducing respiration, ammonium buildup, and browning enzymes such as PPO and LOX. Importantly, antioxidant capacity remained high throughout storage, preventing oxidative deterioration. These findings highlight how nanoparticle-based edible coatings can act simultaneously as physical barriers and biochemical stabilizers, making them particularly suited for protecting MAPs whose value depends on delicate bioactive constituents [147]. These strategies also align with consumer expectations, since the coatings are biodegradable, edible, and often invisible, offering protection without altering appearance or safety.

### 4.3. Nanostructured Polymeric Systems in Crop Protection

Nanocapsules made of chitosan, PLA, or zein are particularly suited to volatile essential oils. They reduce premature evaporation, protect oils from oxidation, and enable release that can be triggered by environmental stimuli such as pH changes or humidity. A notable example of nanostructured systems for plant protection is provided by chitosan nanoparticles encapsulating *Cymbopogon martinii* essential oil (Ce-CMEO-NPs). These nanoparticles, typically 450–480 nm in size with stable surface charge, offered a controlled release of the volatile oil, extending its antifungal efficacy well beyond that of the free compound. When applied against *Fusarium graminearum*—a pathogen notorious for post-harvest losses and mycotoxin production—the nanocapsules achieved superior inhibition of fungal growth and dramatically lowered accumulation of deoxynivalenol (DON) and zearalenone (ZEA) in maize during storage. Mechanistic studies revealed that the encapsulated oil induced oxidative stress and disrupted fungal membranes through lipid peroxidation and ergosterol depletion. Importantly, the nanostructured formulation reduced the concentration of essential oil required for effective control, demonstrating how polymeric nanocarriers can stabilize volatile bioactives, prolong their release, and enhance antifungal potency in real crop storage conditions [148]. The principle can be extended to other volatiles such as eugenol from clove or citral from lemongrass, creating broad-spectrum protective systems, active against a wide range of pathogens, including those particular for MAPs cultivation.

Hydrogels—cross-linked networks of chitosan, starch, or cellulose derivatives—act as reservoirs for both water and active compounds. In arid cultivation areas, hydrogels improve soil moisture retention, buffering plants against drought stress and supporting stable secondary metabolite production [64]. At the same time, nanogels can be applied to foliage, where they adhere to leaves and release fungicides or insecticides slowly, combining abiotic stress management with direct pest control [149].

Electrospinning produces nanofiber mats of polymers like chitosan or PLA that can be impregnated with essential oils [150]. These mats can function as breathable, biodegradable packaging for stored MAP biomass, releasing volatiles gradually to deter fungi or insects. For example, cellulose nanofiber (CNF) coatings demonstrated the possibility of nanostructured polymers to confer passive, yet effective, protection against plant pathogens. In soybean, spraying leaves with a 0.1% CNF suspension shifted surface properties from strongly hydrophobic (~128° contact angle) to hydrophilic (reduced to ~70–90°), which in turn significantly inhibited pre-infection structure formation—germ-tubes and appressoria—by the rust fungus *Phakopsora pachyrhizi* Syd. & P. Syd., (1914). This physical alteration reduced lesion formation and lowered pathogen gene expression related to cell-wall formation (chitin synthases). Importantly, the effect stemmed from the hydrophilic surface per se—not immune priming—as defense-related gene expression was not induced in CNF-treated leaves. This study exemplifies how amphiphilic nanostructured materials can intervene at the earliest stages of infection by modifying host surface microenvironments, offering an eco-conscious, non-toxic route to disease defense [151].

As another example of nanostructured platforms application, electrospun cellulose diacetate nanofiber seed coatings represent a compelling delivery system. In soybean trials, these nanofibrous layers—loaded with model actives like abamectin or fluopyram—did not compromise seed germination and remained physically stable for over two weeks. Their high surface area enabled sustained release of actives; notably, abamectin released more slowly due to its greater hydrophobicity. The practical effectiveness of this approach was demonstrated in vitro, where fluopyram-loaded nanofibers inhibited *Alternaria lineariae*, a common fungal pathogen. This method exemplifies how electrospun polymeric nanofibers can serve as biodegradable, seed-targeted delivery systems, offering sustained, localized protection while reducing chemical runoff and environmental impact [152].

Such approaches open new possibilities for MAP protection that combine biodegradability, efficiency, and multifunctionality. While each of these case studies demonstrates a specific application—whether it is encapsulating essential oils to suppress fungal growth, using cellulose nanofibers to alter surface hydrophobicity, or electrospun nanofibers to coat seeds—the common thread is that nanostructuring transforms polymers from passive barriers into active and intelligent delivery platforms. Instead of merely shielding MAPs from stress, these systems interact dynamically with their environment: they can release biocides only when triggered by humidity, provide sustained antimicrobial action over weeks rather than days, or even modify the plant microenvironment in ways that inhibit pathogen colonization without introducing foreign chemicals. This ability to fine-tune release kinetics, surface interactions, and responsiveness reflects a paradigm shift. Polymers are no longer conceived as inert protective layers but as smart interfaces between plants, pathogens, and the environment. In the context of MAP cultivation—where the purity and stability of bioactives are paramount—this transition toward multifunctional, responsive materials is particularly valuable, as it aligns technical performance with consumer expectations for “clean”, sustainable production.

Representative examples of these nanostructured polymeric systems—ranging from chitosan nanocapsules for essential oils to cellulose nanofibers and electrospun seed coatings—are summarized in Table 4.

### 4.4. Circular Bioeconomy Aspects

A further advantage of polymer-based MAP protection lies in its integration with the circular bioeconomy. Many functional polymers can be derived from agricultural or forestry residues—lignin from pulping, starch from food byproducts, cellulose from crop residues [153]. Instead of being wasted, these materials can be transformed into biopolymeric carriers or coatings. Lignin nanoparticles, for instance, provide not only a renewable encapsulation medium but also intrinsic antioxidant and UV-blocking properties [154], which could enhance the stability of essential oils during storage.

From a sustainability perspective, these systems help reduce reliance on synthetic pesticides and non-degradable plastics. By replacing them with biodegradable, bio-based, and efficient formulations, MAP production can lower its ecological footprint—fewer residues in soil and water, lower greenhouse gas emissions from repeated applications, and less plastic waste. Importantly, the consumers of MAP-derived products are often among the most environmentally conscious—organic food, herbal medicine, and natural cosmetics. In this market, sustainability is not only a responsibility but also a competitive advantage.

### 4.5. Perspectives

Polymers—especially bio-based and nanostructured systems—are redefining how we protect medicinal and aromatic plants. They offer a suite of solutions that are technically effective, environmentally sustainable, and socially aligned with consumer expectations. But their adoption also raises new questions. How do we ensure that polymer residues themselves do not accumulate in soil? How do these materials age in real field conditions? And what are the regulatory pathways for introducing biodegradable polymeric carriers into agro-food systems?

Addressing these questions will require interdisciplinary collaboration. Agronomists, polymer chemists, nanotechnologists, and socio-economists must work together to evaluate not only efficacy but also life-cycle impacts and market acceptance. Advances in digital agriculture, such as precision spraying guided by sensors, may further optimize the use of polymeric systems, reducing inputs while enhancing targeted delivery.

The broader regulatory and policy landscape also shapes the future of polymeric systems for MAP protection. The European Union’s Farm-to-Fork Strategy and the Biodiversity Strategy for 2030 explicitly call for a 50% reduction in pesticide use and associated risks by 2030 [155]. In this context, biodegradable polymer-based carriers for natural bioactives directly contribute to meeting policy targets while maintaining agricultural productivity. Similarly, the European Green Deal emphasizes sustainable agriculture and circular economy principles, further encouraging the adoption of renewable, biodegradable polymers derived from agro-residues. On the market side, certification schemes for organic agriculture and eco-labels for cosmetics and food products place strict limits on synthetic pesticide residues. Polymeric encapsulation of essential oils, edible coatings, and seed-targeted delivery systems can help growers comply with these certification frameworks while safeguarding crop value. Linking research innovation to these policy and certification drivers is therefore essential, not only to accelerate adoption but also to ensure that polymeric solutions move beyond experimental trials and become integral to future MAP value chains.

Ultimately, polymer science provides not only a technical toolkit but a philosophy of protection: protecting plants in ways that are themselves protective of ecosystems, consumer health, and cultural traditions. For MAPs—plants that connect agriculture with medicine, cuisine, and ritual—this alignment of science with sustainability is especially powerful.

## 5. Polymeric Systems for Environmental Protection: The Convergence Point

In the previous sections, was presented the way polymers are helping to protect fragile cultural treasures and high-value medicinal and aromatic plants (MAPs). But if we zoom out, both of these fields are connected by something larger: the state of the environment itself. Cultural assets weathered by pollution, and crops weakened by pesticide residues or soil degradation, are not separate problems but part of the same continuum. They reflect how human activity and climate change are reshaping the ecosystems in which both heritage and agriculture are embedded.

In this broader context, polymer science must show its full versatility. On the one hand, conventional plastics are major contributors to pollution, leaving behind mountains of persistent waste and microplastics that circulate through air, soil, and water [156]. On the other hand, innovative biodegradable, bio-based, and nanostructured polymers are emerging as tools for remediation, ecosystem restoration, and sustainable resource management [157]. Few materials illustrate the paradox of the Anthropocene as clearly as polymers: they are both a major environmental problem and, increasingly, a key part of the solution.

This section explores three interlinked dimensions of the environmental role of polymers. First, we look at the risks common to both heritage and MAP cultivation. Second, we consider how polymers are being engineered to mitigate those risks. Finally, we highlight the shared design principles that position polymer science as a unifying platform for sustainability across domains.

### 5.1. Environmental Risks Common to Heritage and MAPs

Although cultural artifacts and medicinal plants may seem worlds apart, they face surprisingly similar environmental stressors.

#### 5.1.1. Microbial Colonization

Microbes represent one of the most pervasive threats across domains. On frescoes, manuscripts, or stone monuments, fungal and bacterial growth can trigger discoloration, surface erosion, and loss of detail [158,159]. The same fungal spores, when they find their way into dried chamomile or stored lavender, can spoil entire batches, sometimes producing dangerous mycotoxins [160,161]. In both cases, microbial colonization thrives under high humidity and moderate temperatures—conditions increasingly common as climate change disrupts local microclimates. This illustrates how environmental instability magnifies vulnerabilities shared by heritage and crops.

#### 5.1.2. Airborne Pollutants

Urban pollution is another common denominator. Cultural heritage sites exposed to sulfur dioxide, nitrogen oxides, or fine particulates often suffer from accelerated weathering: black crusts on stone, pigment fading, or metal corrosion [162,163]. Medicinal plants exposed to the same pollutants show reduced photosynthesis and slower growth [164]. When particulates settle on leaves, they can contaminate essential oils or extracts, undermining both safety and marketability [165]. Heavy metals carried by dust or smog may even accumulate in harvested biomass, creating risks for pharmaceutical or food use [166].

#### 5.1.3. Climate-Induced Degradation

Climate change is a stress multiplier. For museums and archives, fluctuating temperature and humidity destabilize indoor microclimates, accelerating the aging of both artifacts and conservation polymers [167]. For MAPs, irregular rainfall, droughts, and heat waves reduce biomass and alter the delicate biosynthetic pathways responsible for essential oils [168]. A lavender crop may still flower under stress, but the oil composition might shift away from the desired linalool–linalyl acetate balance, lowering its commercial value. Both heritage and MAPs therefore sit at the frontline of climate instability, bearing silent testimony to the fragility of human and natural systems alike.

#### 5.1.4. Pesticide Residues and Plastic Contamination

Finally, human interventions themselves generate environmental risks. Intensive agriculture relies on pesticides and plastic films, which leave residues in soils and waterways. Pesticide residues disrupt microbial communities and can enter the food chain [169]. Plastic mulches and irrigation pipes fragment into microplastics, which persist in soils for decades, altering water retention and soil fertility [170]. Conservation laboratories are not immune: cleaning agents, synthetic consolidants, and polymeric waste streams also contribute to plastic contamination. These shared risks remind us that heritage and MAPs are not isolated from the wider environment—they are embedded in and dependent on its integrity.

Together, these factors show that the environmental threats facing heritage and MAPs are facets of the same ecological reality. Addressing them requires materials and strategies designed with both protection and sustainability in mind

### 5.2. Polymers as Environmental Protectors

While conventional plastics have contributed heavily to environmental degradation, new generations of polymers are being designed explicitly as protectors of ecosystems.

One of the most promising uses of polymers in environmental protection lies in remediation. Functionalized adsorbent polymers—often based on natural backbones such as chitosan or cellulose—can capture heavy metals and organic pollutants from soil and water. For example, cross-linked chitosan gels can be used for the efficient removal of heavy metals from contaminated water sources [171]. An example on the application of polymers as environmental protectors comes from the development of eco-friendly physically crosslinked hydrogels. Tang et al. [172] reported the design of a chitosan/sodium alginate/calcium ion double-network hydrogel (CTS/SA/Ca^2+^ PCDNH), prepared without toxic chemical crosslinkers. By combining a semi-dissolution acidification sol–gel transition with internal gelation, the researchers obtained a robust hydrogel with markedly improved mechanical strength compared to traditional single-network systems. The resulting material displayed a highly porous three-dimensional structure with abundant hydrophilic groups, providing both high water uptake (swelling ratios up to 110 g/g) and extensive active sites for pollutant capture.

In adsorption studies, the hydrogel demonstrated excellent removal efficiencies for heavy metal ions, achieving adsorption capacities of 176.5 mg/g for Pb^2+^, 70.8 mg/g for Cu^2+^, and 81.3 mg/g for Cd^2+^, surpassing many existing physical hydrogels. Mechanistic analysis showed that Pb^2+^ and Cd^2+^ ions were primarily captured through electrostatic interactions with carboxylate groups, while Cu^2+^ ions also engaged in coordination interactions with nitrogen atoms of chitosan. Importantly, the process was spontaneous and chemophysical in nature, with adsorption controlled by both diffusion and reaction kinetics. The negative Gibbs free energy values further confirmed the feasibility of this approach under environmentally relevant conditions.

From a sustainability perspective, the absence of chemical crosslinkers eliminates risks of secondary contamination, while the biodegradability of chitosan and alginate ensures environmental compatibility. These findings highlight how natural polymer-based hydrogels can simultaneously address pollution remediation (removing toxic heavy metals from water and soils) and align with eco-design principles. Such systems could be particularly relevant in agricultural landscapes where MAPs are cultivated, offering a way to restore soils degraded by metal contamination while reducing reliance on synthetic remediation technologies

In conservation, similar gels have been employed to clean atmospheric soot from stone or marble surfaces, simultaneously preserving heritage and reducing pollutant loads. For example, an agar gel (derived from natural seaweed polysaccharides), was used as a highly effective, sustainable cleaning system for heritage surfaces exposed to pollution. Applied to marble sculptures in the Duomo of Milan, agar gels efficiently removed gypsum, nitrates, soot particles, and dust deposits without damaging the substrate [173]. Compared to conventional water-based cleaning, agar gels offered higher pollutant removal, minimized esthetic alteration, and allowed better control of the cleaning process. The gels also proved safe for operators and visitors, relying on a biodegradable, non-toxic, and low-cost raw material. Optimal results were obtained with 3% agar gel enriched with 1% Tween 20, which enhanced detachment and reduced operation time. Colorimetric and ESEM-EDX analyses confirmed a significant reduction of sulfur (gypsum residues reduced by ~4-fold) and restoration of marble brightness, while preserving surface details.

Another area where polymers can play a protective role is in soil management. Mulching films made from PLA, starch blends, or cellulose derivatives reduce erosion, conserve soil moisture, and suppress weeds—functions traditionally performed by polyethylene mulches [76]. The difference is that biodegradable films decompose safely, without leaving behind plastic residues. For MAP cultivation in arid or semi-arid climates, this is particularly valuable: mulches conserve scarce water and stabilize soil temperatures, helping plants maintain secondary metabolite production even under stress [174]. Here, polymers support not only yields but also environmental sustainability, reducing plastic waste and lowering agriculture’s carbon footprint.

A newer and more experimental frontier is the design of self-degrading polymers with pre-programmed lifetimes. These materials perform their protective role and then disintegrate under specific triggers, such as light, heat, or microbial activity [175,176]. Although this approach is more studied for specific biomedical applications, their use could also expand other areas. In heritage, such coatings could provide temporary protection during transport or restoration, disappearing without residues once the protective window has passed. In agriculture, self-degrading carriers could deliver nutrients or biopesticides gradually, before breaking down harmlessly into the soil. Such systems embody the very principle of “designing for disappearance,” avoiding accumulation and aligning functionality with ecological cycles.

Together, these examples illustrate how polymers can move from being pollutants themselves to becoming active agents of environmental repair and resilience.

### 5.3. The Bridge Between Domains

At the heart of this transition lies a set of shared design principles that cut across heritage, agriculture, and environmental protection.

A decisive shift is underway from petrochemical-based polymers to materials derived from renewable resources like chitosan, cellulose, and PLA. These polymers not only fulfill technical roles but also return safely to natural cycles once their job is performed. This reduces the long-term risks of incompatibility in heritage, minimizes soil contamination in agriculture, and lowers the environmental burden of persistent plastics.

Across applications, polymers must be safe for people and ecosystems. This means avoiding harmful byproducts, ensuring compatibility with the materials they protect, and integrating naturally with biological systems. A striking example is the use of chitosan nanoparticles carrying essential oils: the same formulation can suppress microbial growth on mural paintings or for food preservation, offering protection without toxic residues [177,178].

Perhaps the most exciting frontier is the creation of multifunctional polymeric nanocomposites, combining several roles in a single system [179]. For MAPs, similar systems can act simultaneously as soil conditioners, nutrient carriers, and antifungal agents [180]. This multifunctionality reflects not only technical ingenuity but also an efficiency aligned with circular economy principles—fewer materials, fewer interventions, broader benefits.

By adhering to these principles, polymer science is evolving into a truly integrative discipline—one that addresses not just isolated challenges but the interconnected vulnerabilities of heritage, agriculture, and ecosystems.

### 5.4. Polymeric Systems—The Convergence Point

Polymers hold a curious place in our environmental story. On one hand, they are blamed for many of the crises we face today—plastic waste, microplastic pollution, the long-lived residues that burden soils, rivers, and oceans. On the other hand, they are also being reinvented as some of our most promising allies in the shift toward sustainability. This tension makes polymers more than just materials: they have become symbols of the larger balance between human ingenuity and ecological responsibility.

The convergence of cultural heritage, medicinal and aromatic plants (MAPs), and environmental protection shows that these fields are not isolated at all. They face many of the same threats—microbial colonization, pollutant exposure, climate-induced stress—and increasingly they are finding solutions in the same family of materials. The very same biodegradable chitosan nanoparticle that protects lavender crops from fungal attack can also slow microbial growth on a fresco or remove heavy metals from contaminated water. What may look like coincidence is in fact the outcome of shared design principles: biodegradability, compatibility, multifunctionality, and responsiveness.

This shift is not only technical but also conceptual. Traditional coatings or mulches were designed as passive barriers—they blocked water, sealed surfaces, or delayed evaporation [174]. Modern polymer systems, by contrast, act more like active participants. They can sense changes in humidity or pH, release antimicrobial compounds when conditions demand, or break down on their own once their protective job is performed [181]. This move from inert shield to intelligent interface is perhaps the most profound change in how we imagine the role of materials in both culture and agriculture.

Of course, this transformation must reckon with public perception. For many, “polymer” still means “plastic waste”, and overcoming this association will require more than clever chemistry. Especially in sensitive domains like heritage conservation or organic farming, acceptance depends on trust: polymers must be demonstrably safe, biodegradable, and aligned with sustainability goals. Here, communication matters as much as performance. Showing that these new materials leave no toxic residues, that they degrade into harmless compounds, that they can even be derived from agricultural byproducts—all of this helps to recast polymers from villains into partners in ecological care.

Policy frameworks reinforce this trajectory. The EU Green Deal and Farm-to-Fork Strategy call explicitly for reductions in both pesticide use and plastic pollution, while heritage charters by UNESCO and ICOMOS stress sustainability and reversibility. When polymer science aligns with these priorities, adoption becomes not just easier but almost inevitable. Biodegradable mulches for MAP cultivation directly help meet pesticide-reduction goals; reversible, bio-based coatings in conservation satisfy the ethical principles of heritage preservation. In this sense, science and policy can reinforce each other, pushing polymers from prototypes into mainstream practice.

History, however, reminds us to be cautious. The polymers hailed as breakthroughs in the 1960s—acrylics, epoxies—are now infamous for their irreversibility and long-term degradation [182,183]. To avoid repeating this mistake, future polymer design must include life-cycle thinking from the outset. What byproducts remain after degradation? How do nanoparticles interact with soil microbiomes or aquatic systems? These questions cannot be deferred until after adoption; they must guide the creative process itself.

Perhaps most importantly, the convergence point highlights the need for transdisciplinarity. Polymer chemists alone cannot solve these challenges. They must work alongside conservation scientists, agronomists, environmental engineers, economists, and ethicists. A conservation coating that looks ideal in the lab may fail if museums cannot afford it; a biodegradable mulch may falter if farmers see no economic benefit. Predictive modeling of polymer aging, coupled with socio-economic studies on farmer adoption or museum decision-making, can ensure that solutions are not just scientifically sound but practically viable.

In this light, polymers at the convergence point become more than technical fixes. They embody a new material ethic, one that moves away from the linear “use and dispose” model and toward a circular, responsible material culture. Every intervention—whether on a centuries-old mural or a lavender field—must be designed not only for immediate performance but also for long-term ecological harmony.

**Figure 2:**
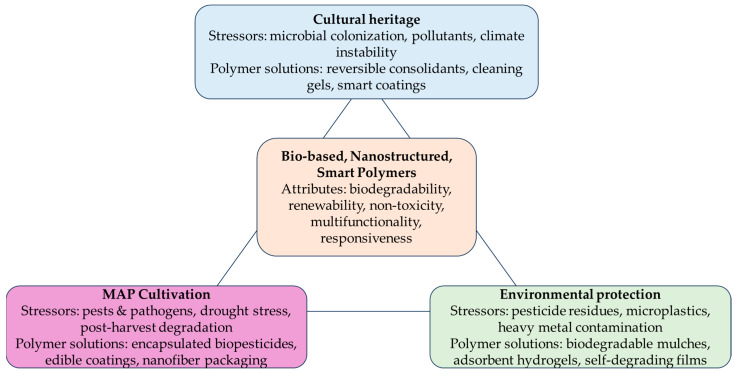
Polymers at the convergence point of cultural heritage, MAP cultivation, and environmental protection. Bio-based and nanostructured polymers emerge as unifying solutions across domains, sharing core principles of biodegradability, renewability, multifunctionality, and responsiveness.

Ultimately, polymers at this convergence point symbolize a deeper cultural choice: how societies reconcile innovation with responsibility. Their future will be defined not just by chemistry, but by whether we embed them in ethical frameworks, policy agendas, and systems of public trust. If performed well, polymers will no longer be seen merely as tools of convenience or pollutants of last resort. They can instead become the connective tissue linking heritage preservation, sustainable agriculture, and environmental stewardship—the very foundations of resilience in the twenty-first century (Figure 2).

## 6. Transdisciplinary Synergies and Future Perspectives

Polymer science for cultural heritage, medicinal and aromatic plant (MAP) protection, and environmental sustainability has come a long way in recent decades. Yet, as the previous sections have shown, the greatest promise does not lie in pursuing incremental refinements within isolated silos. The real frontier emerges where these domains intersect. It is at this intersection that we find a common set of challenges, a growing toolkit of technological strategies, and a horizon of innovation shaped not only by scientific creativity but also by societal values and policy imperatives.

This section reflects on those synergies, explores the technological convergence that is already happening, and looks forward to the research and innovation opportunities that may define the next generation of polymeric systems.

### 6.1. Shared Scientific Challenges

A defining feature of transdisciplinary research is the recognition that distinct application domains frequently converge around structurally analogous scientific challenges. Cultural heritage conservation, the cultivation of medicinal and aromatic plants (MAPs), and environmental protection exemplify this convergence in a particularly striking way.

One such shared challenge is the control of polymer lifetime. In cultural heritage conservation, materials must satisfy the dual requirements of long-term stability and reversibility. A consolidant or coating is expected to provide mechanical reinforcement and chemical stability over decades, yet remain removable to allow future re-treatment as analytical methods and conservation philosophies evolve. This necessitates polymers whose degradation kinetics can be modulated to ensure functionality during their service life, while preserving solubility or degradability pathways compatible with reversibility.

In agricultural systems, by contrast, persistence is advantageous only within a defined temporal window. Polymeric carriers for pesticides, fertilizers, or bioactive compounds must maintain structural integrity long enough to achieve controlled release, but subsequently undergo complete and non-toxic degradation in soil. In this context, kinetic modeling of hydrolytic, enzymatic, or photo-oxidative degradation processes is central to designing carriers that avoid long-term environmental accumulation.

At first glance, these requirements—durability coupled with reversibility in heritage conservation and programmed transience in agriculture—may appear contradictory. However, they are better understood as different expressions of the same fundamental challenge: engineering polymers with precisely controlled degradation profiles. Advances in stimuli-responsive materials, nanostructured carriers, and predictive computational modeling (e.g., digital twins of degradation pathways) now make it possible to tailor polymer lifetimes across a continuum, from weeks to decades, in alignment with the functional and ethical imperatives of each application domain.

This challenge grows sharper at the nanoscale, where degradation rates, release profiles, and safety issues are tightly coupled. A polymer that degrades too slowly may accumulate as microplastics [184]; one that degrades too quickly may fail to deliver its protective role. Designing for “functional time windows” is therefore emerging as a unifying research goal across domains.

Another parallel lies in ethical and regulatory frameworks. In conservation, ethics emphasize reversibility, minimal intervention, and non-toxicity [185]. In agriculture, regulations demand toxicological safety, environmental compatibility, and often rigorous approval pathways for nanocarriers [186]. Both fields therefore grapple with the same tension: how to prove that innovative materials can be both effective and safe, not only in the short term but across decades of use or exposure. For instance, a chitosan nanoparticle carrying thymol might be equally suited for inhibiting fungal growth on frescoes or protecting a thyme field, but its adoption in either context hinges on transparent, harmonized safety data.

These parallels across heritage, agriculture, and environmental protection are not accidental. They reveal that all three fields are, in essence, wrestling with how to reconcile functionality with responsibility. Table 5 summarizes these shared challenges and the convergent polymer-based strategies that may address them, in the authors’ opinion.

### 6.2. Technological Convergence

While shared challenges can appear daunting, they also create fertile ground for technological convergence. Advances in one sector increasingly spill over into others, sometimes with little adaptation required. Nanocellulose offers a telling example. First investigated as a consolidant for fragile paper and textiles [187], it has since been applied as a hydrogel for cleaning stone [119], as a soil conditioner, and even as an encapsulation matrix for bioactive compounds in MAP cultivation [188]. Its high surface area, tunable rheology, and chemical affinity with cellulose-rich substrates make it a true “cross-domain material,” equally at home in archives, fields, or laboratories.

Chitosan shows a similar versatility. As an antimicrobial coating, it protects manuscripts against biodeterioration; as a nanoparticle carrier, it stabilizes volatile essential oils in lavender or oregano fields. The fact that one material can address both conservation ethics and agricultural demands illustrates the power of thinking beyond disciplinary walls.

Knowledge transfer also flows in two directions. Conservation science, with its meticulous focus on compatibility, reversibility, and long-term stability, has lessons to offer agriculture, where short-term field performance has often been prioritized over ecological afterlives. Conversely, agro-nanotechnology—with its extensive experience in encapsulation, controlled release, and scaling—offers conservationists a roadmap for moving innovations out of the lab and into practical use.

A particularly exciting area of convergence is multifunctional nanocomposites. A perspective in this area could be research for developing multitasking matrixes that consolidate cultural heritage objects, adsorbs atmospheric pollutants, and releases antifungal agents, in the same time. With minor reformulation, the same system could serve as a soil conditioner, nutrient carrier, and antifungal protectant in MAP fields. These multifunctional systems embody efficiency, a trait that aligns with circular bioeconomy principles: fewer inputs, broader impact, and reduced waste.

In short, technological convergence turns polymers into modular platforms. Rather than reinventing solutions for each sector, the same polymer design can be adapted across fields, saving time, reducing costs, and encouraging systemic sustainability.

### 6.3. Research and Innovation Opportunities

Looking forward, the opportunities for polymer science lie at the nexus of science, policy, and society. Several dimensions are worth highlighting.

First, the policy landscape already provides fertile ground. The European Union’s Horizon Europe missions exemplify this: the Heritage mission emphasizes sustainable preservation, the Food mission promotes resilient agricultural systems, and the Environment mission seeks to address pollution and climate adaptation. Polymeric systems that are bio-based, reversible, and multifunctional fit all three agendas simultaneously. A single innovation—a biodegradable nanoparticle carrying an essential oil—can contribute to cultural heritage resilience, pesticide reduction in agriculture, and ecological health. Such cross-mission relevance makes polymer science uniquely positioned to attract interdisciplinary funding and collaboration.

Second, the lack of standardized testing protocols remains a bottleneck. Conservation researchers rely on artificial aging tests, agricultural scientists test biodegradability in soil, and environmental toxicologists run ecotoxicity assays. Without harmonization, results are hard to compare, and regulatory approval is slowed. A promising avenue could be, in our opinion, the development of modular testing platforms that evaluate polymers for durability, biodegradability, reversibility, and ecotoxicity in parallel, creating shared datasets that can support multiple applications at once.

Third, emerging digital tools hold transformative promise. Artificial intelligence (AI) can accelerate the discovery of new blends by mining large datasets on polymer chemistry and performance. Digital twins—virtual models that simulate polymer behavior under different conditions—could predict how a coating performs on parchment, how a hydrogel interacts with lavender roots, and how a nanocomposite ages in polluted air, all before deployment. These tools allow not only faster optimization but also deeper insight into long-term impacts, bridging laboratory studies with real-world performance.

Finally, the social dimension must not be overlooked. Polymers suffer from a reputation problem, often equated with plastic waste and microplastic pollution. To counter this, scientists must communicate clearly how bio-based and nanostructured polymers differ from conventional plastics—demonstrating that when designed responsibly, they are not pollutants but enablers of sustainability. Outreach to cultural institutions, farmers’ cooperatives, and environmental NGOs can ensure that innovation is not just technically feasible but also socially trusted.

### 6.4. Toward a Shared Future

The synergies explored here point toward a future where polymers are no longer categorized as “heritage materials”, “agricultural inputs”, or “environmental technologies”. Instead, they may form part of a shared material culture of sustainability, designed to function across contexts while respecting ecological and ethical principles.

To realize this vision, several priorities deserve emphasis:

- Life-cycle thinking as standard practice. Past mistakes—like the widespread use of acrylics and epoxies—show that materials celebrated as solutions can later become problems. Designing with end-of-life in mind must become the norm. Researchers must ask: what residues remain after degradation? How do nanoparticles interact with soil microbiomes or aquatic ecosystems? Such questions must be answered before large-scale adoption.
- Policy alignment as catalyst. From the EU Green Deal to the Farm-to-Fork Strategy, policy frameworks are moving rapidly toward pesticide reduction, circularity, and green chemistry. Polymer research that aligns with these frameworks will move faster from prototype to practice. For example, biodegradable mulches in MAP cultivation directly contribute to pesticide reduction targets, while reversible coatings align with UNESCO principles for cultural heritage.
- Transdisciplinarity as necessity. Effective progress requires not just collaboration between chemists and conservators, but also agronomists, environmental engineers, economists, and ethicists. Predictive modeling of material aging must be integrated with socio-economic studies of farmer adoption or museum budgets. This ensures that innovations are both technically sound and practically viable.
- Cultural reframing. Polymers must move in the public imagination from being “symbols of pollution” to “symbols of responsible innovation”. Communicating stories of how polymers can protect a medieval manuscript, a lavender field, and a river ecosystem simultaneously can shift perceptions and build public trust.

Ultimately, polymers at this convergence point symbolize more than technical ingenuity. They embody a philosophical transition: away from linear “use-and-dispose” material culture and toward a circular, responsible approach that balances innovation with stewardship. The fact that a single class of materials can serve to conserve the past, protect the present, and safeguard the future is a powerful reminder of what science can achieve when guided by responsibility as much as by creativity.

If pursued wisely, polymers will not only solve immediate challenges but also become the connective tissue linking heritage preservation, sustainable agriculture, and ecological resilience. In doing so, they will help define what it means to build a sustainable society in the twenty-first century.

## 7. Conclusions

Polymers tell a story that mirrors the twentieth and twenty-first centuries. First celebrated as miracle materials, they later became symbols of excess and waste. Today, that story is being rewritten. With the rise in bio-based and nanostructured systems, polymers are no longer seen simply as inert plastics but as designed materials that can protect heritage, support agriculture, and restore the environment—all while respecting ecological boundaries.

What unites these very different fields—cultural heritage, MAP cultivation, and environmental protection—is the search for balance. Conservators want materials that last yet remain reversible. Farmers want crop protection that works in the field but leaves no harmful residues. Environmental scientists want polymers that perform their task and then return harmlessly to nature. These are not contradictions; they are variations in the same challenge: designing materials with lifetimes that are matched precisely to their purpose.

The convergence of these fields is already visible. The same chitosan nanoparticle can keep a fresco free from fungi, protect a lavender crop, or remove pollutants from water. Nanocellulose, first used in archives, now strengthens soils and stabilizes bioactive compounds. These overlaps show that polymers are becoming true “platform materials,” adaptable across contexts rather than confined to one sector.

But technical innovation alone is not enough. Public trust must be earned, especially since “polymer” still evokes the image of plastic waste. The future will depend on proving—through transparency, standards, and dialogue—that new polymer systems are safe, biodegradable, and beneficial. Equally important is policy alignment: when polymer design supports the EU Green Deal, Farm-to-Fork, or UNESCO conservation ethics, it gains both legitimacy and momentum.

The lesson of history is cautionary. Materials once praised for durability—like acrylics and epoxies—became long-term conservation problems. To avoid repeating those mistakes, life-cycle thinking must guide all new designs. What happens when a polymer ages, breaks down, or interacts with living systems must be answered from the outset, not after deployment.

Ultimately, the real shift is cultural. Polymers are being reimagined not as disposable commodities but as part of a circular, responsible material culture. If designed wisely, they can act as connectors: linking past and present by preserving heritage, protecting livelihoods by supporting MAP cultivation, and ensuring resilience by healing ecosystems. In this sense, polymers can become more than tools of science; they can become symbols of how innovation and responsibility can be reconciled in the twenty-first century.

## Figures and Tables

**Table 1:** Comparison of synthetic vs. bio-based polymers.

Domain	Synthetic Polymers (Limitations), [Reference]	Bio-Based Polymers (Advantages), [Reference]
Cultural Heritage	PVAc, acrylics, epoxies (poor reversibility, yellowing, embrittlement) [34,35]	Chitosan, cellulose, PLA (reversible, compatible, biodegradable) [75]
Agriculture	Seed/fertilizer coatings, mulching films (persistence, residues) [76]	Chitosan, starch, PLA (controlled release, biodegradable coatings) [77]
Environmental protection	Durable plastics (accumulation, microplastic pollution) [9]	Cellulose, PLA, proteins (adsorbents, biodegradable films, reduced footprint) [78]

**Table 2:** Emerging nanostructured polymer systems and cross-domain applications.

Nanostructured System	Heritage Science Applications	Agriculture Applications	Environmental Applications
Nanofibers	Breathable protective coatings, antimicrobial materials [92]	Controlled release materials for fertilizers/pesticides [93]	Filtration, pollutant capture materials [94]
Nanocapsules	Deep penetration consolidants [95]	Encapsulation of essential oils, phenolics [96]	Encapsulation of remediation agents [97]
Nanocomposites	Transparent consolidants, reinforcement of fragile materials [98]	UV protection, nutrient release control [99]	Adsorbents, photocatalysts for pollutants [100]
Smart polymers	Humidity/light-responsive protective films [101]	Soil pH/moisture-triggered release systems [102]	Self-degrading plastics, adaptive remediation materials [103]

**Table 3:** Bio-based polymers applied in cultural heritage conservation: requirements addressed, applications, advantages, and challenges.

Polymer/System	Key Requirement(s) Addressed	Example of Application	Advantages	Limitations/ Challenges	Reference
Chitosan (Low-MW—25–45 kDa, 1%)	Antimicrobial protection, compatibility, non-alteration	Added to sturgeon-glue mock layers; inhibited multiple tempera-painting fungal strains	Effective against resistant fungi; did not affect optical/surface properties; biodegradable; reversible	Requires molecular weight optimization; needs real-artwork validation	[60]
Bacterial Nanocellulose (BC) hydrogel loaded with EDTA	Cleaning capability, compatibility, reversibility, sustainability	Green cleaning of copper stains on marble using BC hydrogels loaded with 1% *w*/*v* EDTA applied for 120 min	Biodegradable; high cleaning efficacy; chromatically safe (ΔE* < 5); peelable with no residue	Effective only with ≥1% EDTA and sufficient application time	[119]
Proteins (gelatin nanolayer/nanoparticles)	Compatibility, micro-scale consolidation	Stabilization of damaged gelatin photographic emulsions	Compatible with original binder; targeted consolidation	Requires characterization of long-term behavior	[120]
Proteins (self-regenerated silk fibroin films)	Mechanical reinforcement, compatibility, sustainability	Applied to aged silk textiles; tunable crystallinity/amorphousness restored ductility and strength	Fully compatible with silk; sustainable (waste silk source); tailored mechanical properties; reversible	Performance depends on fibroin concentration; requires optimization for each degradation state	[121]
Starch	Reversibility, flexibility, chemical stability	Paper and book conservation; hinging, mending, leafcasting, wall paintings	Durable yet flexible; reversible with moisture; chemically stable; simple proteome reduces risk of long-term alteration	Susceptible to dehydration (contraction, fracture); lower adhesion than flour pastes; potential microbial vulnerability	[125]
PLA/MMT bio-nanocomposite coatings	Hydrophobicity, durability, environmental sustainability, reversibility	Applied to Marmara marble to reduce water infiltration and pollution-induced sulfation	Increased water contact angle (from ~75° uncoated to 108° with PLA/MMT5); reduced capillary absorption by 46–66%; decreased water vapor transmission up to 59%; improved resistance to SO_2_-induced gypsum crust formation (4–5 × less than uncoated); preserved marble color at ≤5 wt% clay; biodegradable, reversible	At 7 wt% clay, poor dispersion caused color change (ΔE > 3) and reduced performance; requires optimization of filler concentration and long-term aging validation	[70]

**Table 4:** Representative bio-based and nanostructured polymeric systems applied for plant protection.

Polymer System	Encapsulated/Active Agent	Target/Stressor	Application Mode	Key Outcomes	Reference
Chitosan nanoparticles (CSNPs)	Thymol	*Xanthomonas campestris* (Pammel 1895) Dowson 1939 (bacterial pathogen)	Foliar application	Eliminated bacteria at 500 µg/mL within 24 h; caused membrane disruption, ROS production, lipid peroxidation, and metabolic damage (lipids, proteins, nucleic acids).	[143]
Chitosan nanoparticles (CSNPs)	Carvacrol	*Candida* spp. (fungal model; antifungal protection)	In vitro assays (planktonic and biofilm)	Potent inhibition of planktonic growth, strongest against *C. tropicalis* and *C. krusei*; encapsulation improved antifungal efficacy compared to free carvacrol.	[144]
Chitosan nanoparticles (CSNPs) edible coating	— (coating film)	Post-harvest deterioration of coriander leaves	Edible coating	Extended shelf life >2×; preserved chlorophyll, carotenoids, proteins, and essential oils; reduced respiration, PPO/LOX activity; maintained antioxidant activity.	[147]
Chitosan nanoparticles (Ce-CMEO-NPs)	*Cymbopogon martinii* essential oil	*Fusarium graminearum* (fungal pathogen, mycotoxin producer)	Post-harvest storage of maize (model)	Nanoparticles (~450–480 nm) provided controlled EO release; superior fungal inhibition; reduced DON and ZEA mycotoxin accumulation; lowered EO dosage required.	[151]
Cellulose nanofibers (CNF)	— (surface coating)	*Phakopsora pachyrhizi* Syd. & P. Syd., (1914) (soybean rust fungus)	Foliar spray (0.1% CNF suspension)	Shifted leaf surface from hydrophobic to hydrophilic; inhibited germ-tube/appressoria formation; reduced lesions without inducing plant immunity.	[152]
Electrospun cellulose diacetate (CDA) nanofibers	Abamectin or fluopyram	Pests (abamectin) and fungal pathogens (fluopyram vs. Alternaria *lineariae*)	Seed coating (soybean)	Nanofiber coatings stable >2 weeks; no effect on germination; sustained AI release; fluopyram-loaded fibers suppressed fungal growth effectively.	[115]

**Table 5:** Shared scientific challenges across domains and convergent polymer-based solutions.

Domain	Shared Challenge	Polymer-Based Solution	Cross-Domain Relevance
Cultural Heritage	Need for stability but also reversibility of treatments	Bio-based consolidants (e.g., chitosan, nanocellulose) with reversible solubility	Informs agriculture on designing carriers that persist during use but degrade safely
MAP Cultivation	Need for persistence in the field but no harmful residues	Controlled-release biodegradable carriers (PLA, starch, alginate)	Inspires conservation to use “time-limited” coatings or delivery systems
Environment	Pervasive pollution from persistent plastics and residues	Biodegradable mulches, adsorbent gels, self-degrading films	Encourages heritage and agriculture to adopt life-cycle design principles
All domains	Regulatory approval and ethical acceptance	Non-toxic, renewable polymers; standardized safety tests	Supports harmonization of regulations across heritage, food, and environment
All domains	Unpredictable performance under real conditions	AI modeling and digital twins for degradation and compatibility	Accelerates testing and adoption across multiple contexts

## Data Availability

No new data were created or analyzed in this study. Data sharing is not applicable to this article.

## Abbreviations

The following abbreviations are used in this manuscript:

AI	Artificial Intelligence
BC	Bacterial Nanocellulose
CDA	Cellulose Diacetate
CMC	Carboxymethyl Cellulose
CNC	Cellulose Nanocrystals
CNF	Cellulose Nanofibrils
CSNPs	Chitosan Nanoparticles
Ce-CMEO-NPs	Chitosan Nanoparticles loaded with *Cymbopogon martinii* Essential Oil
DON	Deoxynivalenol
ESEM-EDX	Environmental Scanning Electron Microscopy with Energy Dispersive X-ray Spectroscopy
EU	European Union
FTIR	Fourier Transform Infrared Spectroscopy
HPC	Hydroxypropyl Cellulose
ICOMOS	International Council on Monuments and Sites
LNPs	Lignin Nanoparticles
LOX	Lipoxygenase
MAPs	Medicinal and Aromatic Plants
MMT	Montmorillonite
NGO	Non-Governmental Organization
PCDNH	Physically Crosslinked Double-Network Hydrogel
PHA	Polyhydroxyalkanoates
PLA	Polylactic Acid
PPO	Polyphenol Oxidase
PVAc	Poly(vinyl acetate)
ROS	Reactive Oxygen Species
SDGs	Sustainable Development Goals
SO_2_	Sulfur Dioxide
UNESCO	United Nations Educational, Scientific and Cultural Organization
ZEA	Zearalenone
